# Low let-7d microRNA levels in chick embryos enhance innate immunity against *Mycoplasma gallisepticum* by suppressing the mitogen-activated protein kinase pathway

**DOI:** 10.1186/s13567-023-01178-6

**Published:** 2023-06-19

**Authors:** Yingjie Wang, Huanling Sun, Wenqing Zhao, Tengfei Wang, Mengyun Zou, Yun Han, Yingfei Sun, Xiuli Peng

**Affiliations:** Key Laboratory of Agricultural Animal Genetics, Breeding and Reproduction, Ministry of Education, Huazhong Agricultural University, Wuhan, 430070 Hubei China

**Keywords:** *Mycoplasma gallisepticum* HS strain (MG-HS), chicken primary type II pneumocytes (CP-II), let-7d, MKP1, MAPK pathway

## Abstract

Chick embryos are a valuable model for studying immunity and vaccines. Therefore, it is crucial to investigate the molecular mechanism of the *Mycoplasma gallisepticum* (MG)-induced immune response in chick embryos for the prevention and control of MG. In this study, we screened for downregulated let-7d microRNA in MG-infected chicken embryonic lungs to explore its involvement in the innate immune mechanism against MG. Here, we demonstrated that low levels of let-7d are a protective mechanism for chicken embryo primary type II pneumocytes (CP-II) in the presence of MG. Specifically, we found that depressed levels of let-7 in CP-II cells reduced the adhesion capacity of MG. This suppressive effect was achieved through the activated mitogen-activated protein kinase phosphatase 1 (MKP1) target gene and the inactivated mitogen-activated protein kinase (MAPK) pathway. Furthermore, MG-induced hyperinflammation and cell death were both alleviated by downregulation of let-7d. In conclusion, chick embryos protect themselves against MG infection through the innate immune molecule let-7d, which may result from its function as an inhibitor of the MAPK pathway to effectively mitigate MG adhesion, the inflammatory response and cell apoptosis. This study may provide new insight into the development of vaccines against MG.

## Introduction

*Mycoplasma gallisepticum* (MG) is a notorious pathogen with a high prevalence in poultry worldwide that causes highly contagious chronic respiratory disease (CRD) in chickens [1, 2]. Adhesion of MG is a complex multifactorial process and is a prerequisite for its pathogenicity [3]. MG adheres to avian lungs and triggers a vigorous inflammatory response, leading to lung tissue lesions [4, 5]. The MG-HS strain, a virulent strain, was isolated from Hubei, and pMGA1.2 is a major adhesion protein of MG-HS and is necessary for MG-HS infection in chickens [6]. Worse, our previous studies have demonstrated the ability of MG to evade host immunity through the gga-mir-365-3p/SOCS5/STATs axis [7]. Elimination of the risk of MG infection is a challenge for the poultry industry [8].

Vaccination is currently an effective approach to prevent MG infection. The nonspecific response of innate immunity is a prerequisite for the initiation of the acquired immune response to vaccines [9]. Due to the high variability of strain antigens and the instability of attenuated vaccines, the development of novel vaccines is needed. An in-depth understanding of the innate immunity mechanism of chick embryos against MG facilitates vaccine development.

MicroRNAs (miRNAs) are short endogenous noncoding RNAs that are thought to be important regulators of innate and adaptive immune responses in most eukaryotes [10]. In a previous study, we found that let-7 possibly participated in the innate immune process of chick embryos against MG infection, but its exact mechanism is not yet clear [11]. Let-7, one of the first miRNAs identified, has been shown to be involved in innate immunity to a variety of pathogenic microorganisms. For example, in *Caenorhabditis elegans*, let-7 plays a role in regulating innate immunity in the intestine and neurons [12]; let-7 directly inhibits TLR4, thereby contributing to the initiation of the innate immune response and inflammation during *Helicobacter pylori* or *Cryptosporidium parvum* infection [13, 14].

The mitogen-activated protein kinase (MAPK) signalling pathway, a major effector limb of inflammatory lesions and immune responses, is an attractive therapeutic target [15]. Mitogen-activated protein kinase phosphatase 1 (MKP1) belongs to the bispecific phosphatase family and is an endogenous negative regulator of MAPK signalling [16]. Dephosphorylation of MKP1 suppresses the activated protein-1 (AP-1) signalling pathway, resulting in downregulation of the inflammatory cytokines IL-6, IL-8, IL-12, TNF-*α*, and COX2 [17]. AP-1 consists of a DNA-binding complex of Jun, Fos and the cAMP response element binding the protein family of activated transcription factors [18]. It has been previously demonstrated that inhibition of the MAPK pathway leads to weakened inflammation, thereby activating host innate immune mechanisms. Nevertheless, the mechanism through which let-7d regulates the innate immune response in MG-infected chick embryos remains unclear.

In this study, we identified enrichment of let-7d target genes within the MAPK pathway. As such, we sought to investigate the potential role of let-7d in regulating the innate immune response against MG infection in chick embryos by modulating the MAPK signalling pathway.

## Materials and methods

### Chicken embryo primary type II pneumocyte (CP-II) culture

CP-II cells were prepared using standard techniques from 14-day-old specific pathogen-free (SPF) embryos. Chicken embryonic lungs were cut into tiny tissue pieces and then digested using 0.25% trypsin and 0.1% type IV collagenase (Invitrogen-Gibco) at 37 °C for 15 and 20 min, respectively. The cell suspension was filtered through a 200-mesh sieve and incubated with 10% foetal bovine serum for one hour, and then, the supernatant was collected to remove the adhered cells. The supernatant collected in the above step was centrifuged at 1200 r/min for 5 min to collect the unattached cells. Subsequently, the precipitated cells were resuspended in DMEM and then filtered through a 400-mesh sieve to obtain CP-II cells. The purified CP-II cells were counted and inoculated in appropriate amounts into culture plates in a carbon dioxide cell incubator with 5% CO_2_ at 37 °C [19].

### Mycoplasma strains and infection experiments

MG-HS, a virulent strain of mycoplasma, was isolated from a chicken farm in Hubei Province, China [20, 21]. MG-HS was provided by the State Key Laboratory of Agricultural Microbiology, College of Veterinary Medicine, Huazhong Agricultural University (Wuhan, China). MG-HS was cultured on the basis of a previous study [22]. In brief, MG-HS was cultured at 37 °C in modified FM-4 medium with 12% (v/v) porcine serum and 10% yeast extract until the mid-log phase.

The concentration of viable *Mycoplasmas* in a suspension was then determined by a colour-changing unit (CCU) assay that was reported in detail in our previous studies [23]. Briefly, twenty sterilized 2 mL centrifuge tubes were prepared, and 1.8 mL of medium was added to tubes 1–19. Next, 0.2 mL of the mycoplasma solution was added to the first centrifuge tube and mixed thoroughly. Subsequently, 0.2 mL of the mixture was aspirated into the second centrifuge tube and mixed well. This process was repeated for each subsequent centrifuge tube until reaching the 19^th^ tube, at which point 0.2 mL of liquid was discarded after mixing. The 20^th^ centrifuge tube (tube 20) was designated as the control. The tubes were then incubated at a constant temperature of 37 °C for a duration of two weeks, during which the colour change of the medium was observed.

The concentration of MG-HS in this study was 10^9^ CCU/mL. In addition, White Leghorn specific-pathogen-free (SPF) chick embryos were used for MG-HS infection experiments. The experimental method was detailed in our previous reports [11]. In short, the chick embryo allantoic cavity was injected with 300 µL of MG after 9 days of incubation. Then, samples of chicken embryonic lung tissue were collected at 3–7 days post-infection (equivalent to days 12–16 of egg hatching).

### DNA primers and RNA oligonucleotides

In this experiment, the total sequences of the DNA primers that were adopted are presented in Table 1. In addition, all RNA oligonucleotides were designed and synthesized by GenePharm (Shanghai, China). The RNA oligonucleotide sequences are shown in Table 2.

**Table 1:** **Sequences of DNA primers**.

Name	Primer sequence (5′-3′)
	Primers for CDS Cloning
MKP1-CDS-F	CCCTCGAGATGGTGAACCTGCGGGTGT
MKP1-CDS-R	TTGCGGCCGCTCAGCAGCTCGGGGAGG
	Primers for 3’UTR Cloning
MKP1 3’UTR-F	CCCTCGAGTTGTCCTAAGCCTGCTGCAT
MKP1 3’UTR-R	TTGCGGCCGCGCAACTCAAGAAGCACTCGC
Mut- MKP1 3’UTR-F	AGAGAAACCAAATGATCGAGTATTTTTTTTTTGGTACTGTAATCCTGTGTGT
Mut- MKP1 3’UTR-R	AAATACTCGATCATTTGGTTTCTCTAGAAATGCAACTTCAGGTTCATG
	Primers for RT‒qPCR
GAPDH-F	GAGGGTAGTGAAGGCTGCTG
GAPDH-R	CACAACACGGTTGCTGTATC
MKP1-F	GGCACTACCAGTACAAAAGCATC
MKP1-R	CAAGGACCTGCGACTCGAAC
ERK-F	GTGACTTCGGACTGGC
ERK-R	TCCTGGAAAGATGGGT
P38-F	ACGGCTACTGAAGCATAT
P38-R	TCTGTAAGTTTCTGGCATT
JNK-F	AAGCAAGCGTGACAAC
JNK-R	ATCATAGGCTGCACATAC
C-jun-F	CGGCTCATCATCCAGTCC
C-jun-R	CCCTCTTGCTCGTCGGTA
C-fos-F	TACACCTCCACCTTCGTCTTCACC
C-fos-R	TTGCTGCTGCTGCCCTTCC
CCL4-F	CCTCGCTGTCCTCCTCA
CCL4-R	CTGGCTGTTGGTCTCGTAG
CCL5-F	CTCAAGCCTCTTCATCTCC
CCL5-R	GCATTTGCTGCTGGTGT
PMGA1.2-F	TCATATAAAAACTTAATGGCTACTC
PMGA1.2-R	CTCTTCTGTGATTGTATTAGCAG
RT-Let-7d-F	CTCAACTGGTGTCGTGGAGTCGGCAATTCAGTTGAGACTATGCA
miR-let-7d-F	CTGGTAGGAGAGGTAGTGGGTTGC
miR-let-7d-R	ACTGGTGTCGTGGAGTCGGC
5s-rRNA-F	CCATACCACCCTGGAAACGC
5s-rRNA-R	TACTAACCGAGCCCGACCCT
RT-5 S	AACTGGTGTCGTGGAGTCGGC

**Table 2:** **Sequences of RNA oligonucleotides**.

Name	Sequences (5′–3′)
Si-NC	CAGUACUUUUGUGUAGUACAA
Si-gga-MKP1	GAAAGCAAGTAGTAATTGA

### RNA isolation and quantitative real-time PCR

According to the manufacturer’s instructions, total RNA was isolated from postinfected and noninfected cells using the FastPure® Cell Total RNA isolation kit (RC112-01, Vazyme Biotech Co., Ltd., China). RNA was reverse transcribed to cDNA with the First Strand cDNA Synthesis Kit (Cat No. 11,119–11,141; Yeasen, Shanghai, China), and reverse transcription PCR (RT‒PCR) was performed. The total volume of the reaction was 10 µL, and the thermocycler program was as follows: 94 °C for 10 min and 40 cycles of 94 °C for 20 s, 58 °C for 20 s, and 72 °C for 20 s. The relative mRNA levels were calculated using the 2 − ΔΔCt method [24]. GAPDH was used as an internal control.

### Construction of the 3’-UTR-luciferase plasmid and dual-luciferase reporter assay

The wild-type and mutant 3’-UTR DNA fragments of MKP1 covering the predicted binding sites of let-7d were successfully replicated. The psiCHECK™-2-MKP1-3’-UTR (wild type and mutant) vector was constructed by combining the luciferase vector psi-CHECK™-2 (Promega, Madison, WI, USA) with the MKP1 3’-UTR (wild-type and mutant). The dual-luciferase reporter assay was performed as we previously reported [25].

### Cell transfection

Once 80–90% confluence was achieved, each group of cells was transfected with let-7d-mimics, let-7d-mimic negative control, let-7d-inhibitor, and let-7d-inhibitor negative control. CP-II cells transfected with let-7d-mimics were marked as mimics; cells transfected with let-7d-inhibitor were marked as Inhibitor; and cells transfected with a nonspecific RNA were marked as mimics-NC or Inhibitor-NC. The cells were harvested for subsequent studies 48 h after transfection.

### Cell proliferation and apoptosis assays

Cell Counting Kit-8 (CCK-8, Dojindo, Shanghai, China) assays were used for cell proliferation experiments. CP-II cells were inoculated in a 96-well plate at 2 × 10^4^ cells per well. Each group of cells was separately transfected with different oligonucleotides (let-7d, let-7d-NC, let-7d-inhibitor, let-7d-inhibitor-NC, Si-MKP1, Si-MKP1-NC) or plasmids (pcDNA3.1-empty, pcDNA3.1-MKP1) using Lipofectamine™ 3000, and each group had 6 biological replicates. Next, MG-HS (7 µL, 10^10^ CCU/mL) was utilized to infect CP-II cells for 2 h. At 24 h, 48 h, and 72 h post-transfection, cell proliferation curve was measured by the CCK-8 kit according to the manufacturer’s instructions.

Transfection treatments were described above. An Annexin V FITC apoptosis detection kit (Dojindo) was used to test cell apoptosis. The cell suspension, which was made from logarithmic growth phase CP-II cells, was infected with MG for 2 h and then separately transfected with RNA oligonucleotides according to the instructions of FectinMore™ (purchased from Chamot Biotechnology Co., Ltd.). At 48 h post-transfection, ice-cold phosphate-buffered saline (PBS) was utilized to wash cells once, and harvested cells were centrifuged (2000 r/min, 5 min) and resuspended in binding buffer containing 5 µL of Annexin V-FITC at a density of 1–5 × 10^5^ cells/mL. Cells were then incubated with 5 µL of propidium iodide (PI) for 15 min in the dark. Finally, the apoptosis of CP-II was analysed by fluorescence-activated cell sorting (BD Biosciences). Each group was assayed three times.

### ELISAs

The grouping of transfection treatments was described above. Forty-eight hours after transfection, the supernatants were collected, and the proinflammatory cytokine (IL-6, IL-10, IL-1*β* and TNF-*α*) levels were detected with enzyme-linked immunosorbent assay kits (Bio Legend, San Diego, CA, USA) according to the manufacturer’s directions.

### Western blot

The grouping of transfection treatments was described above. Forty-eight hours after transfection, the total proteins were extracted from CP-II cells, and their concentrations were then determined using a bicinchoninic acid (BCA) protein assay reagent kit (Transgen, Shanghai, China). Equal amounts of protein were separated by 12% SDS-polyacrylamide gel electrophoresis (Beyotime, China) and blocked with 5% skim milk for 1 h. Then, primary antibodies for rabbit anti-MKP1 (ABclonal, A2919), p-ERK (ABclonal, AP0472), p-JNK (ABclonal, A4867), P38 (ABclonal, A0227), GAPDH (Abmart, M20024) or β-actin (Abmart, T40104) (at 1:5000 dilution) protein were incubated overnight at 4 ℃. Finally, the membrane was incubated with secondary antibody for 1 h after Tris-buffered saline with Tween-20 (TBST) (a 1× concentrated solution of Tris-buffered saline with Tween-20 with a concentration of 10 mM Tris-HCl, 15 mM NaCl, 0.05% Tween-20 at pH 7.5) washing. The enhanced chemiluminescence (ECL) detection system (Bio-Rad) was used to detect protein expression.

### Statistical analysis

Three independent duplicates were set in each experimental group, and the results were analysed using GraphPad Prism 7. After one-way ANOVA, multiple comparisons of Tukey’s HSD method were used to compare the different experimental values or Student’s *t* test was used for the comparison of two conditions. The data are expressed as the mean ± SD. *P* values < 0.05 were considered to indicate significant differences.

## Results

### Let-7d expression is decreased in MG-infected CP-II cells

**Figure 1:**
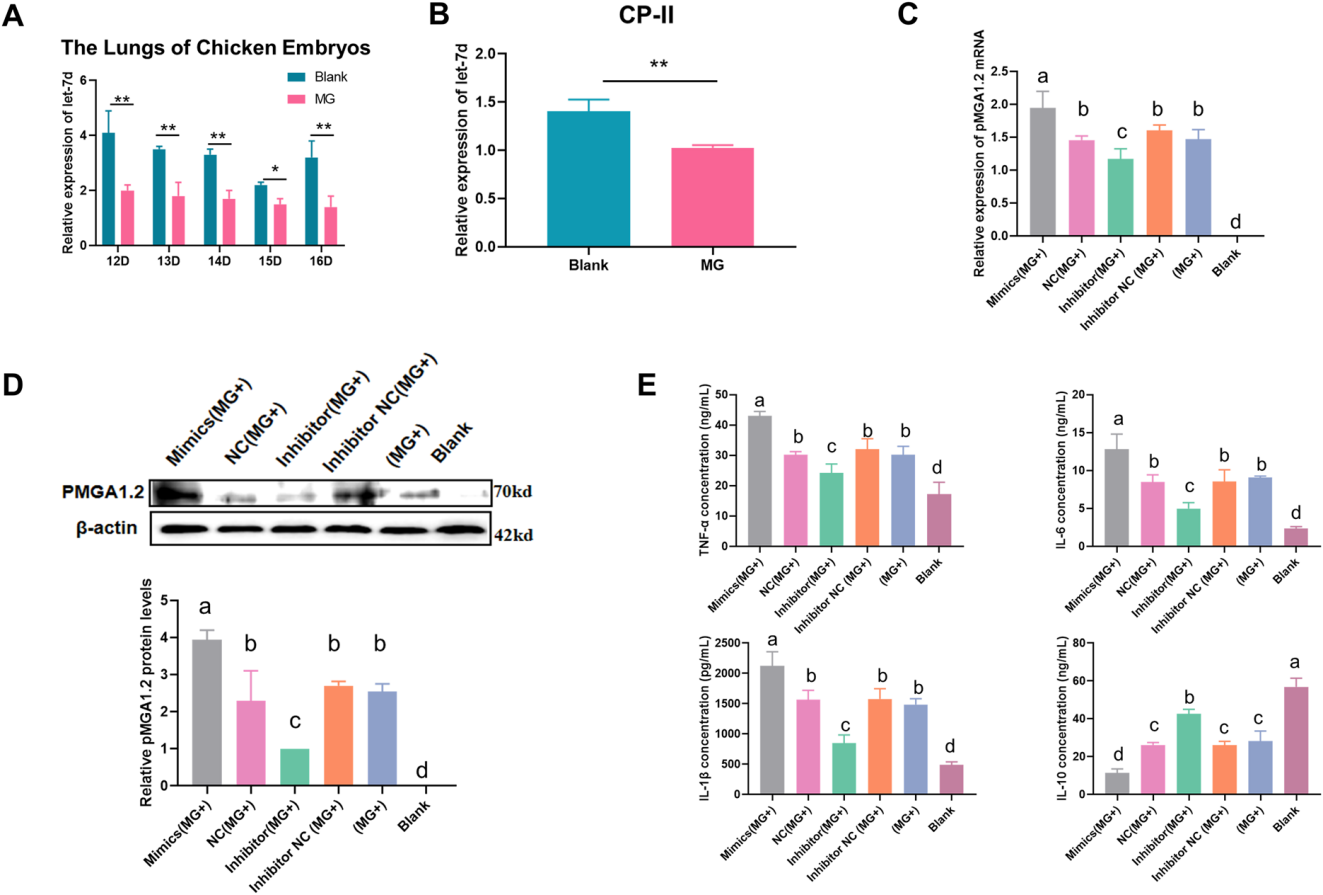
**Relative let-7d expression level in CP-II cells and chicken embryonic lung tissue.** (**A**) The relative level of let-7d in chicken embryonic lungs. (**B**) The relative level of let-7d in CP-II cells. The above data were corrected via 5sRNA as the internal quantitative control gene. (**C, D**) qPCR and WB were used to detect the mRNA or protein expression of pMGA1.2. Positive chicken serum was used as the primary antibody against pMGA1.2. (**E**) Downregulated let-7d regulates inflammatory cytokine production in CP-II cells. The levels of IL-6, IL-1*β*, IL-10 and TNF-*α* were analysed by ELISAs at 48 h post-transfection. All measurements shown are the mean ± SD from three independent experiments, each with three replicates. (*p < 0.05, **p < 0.01). Different lowercase letters represent *p* < 0.05.

First, we used MG-infected CP-II cells as a model to investigate the molecular mechanisms of innate immunity against MG in chick embryos. Our previous miRNA deep sequencing data showed that let-7d was significantly downregulated in MG-infected chicken embryonic lung tissue [11]. In the present experiment, we observed a significant decrease in let-7d expression in the lungs of the MG-infected chick embryos compared to that of the noninfected group on the 3^rd^ to 7^th^ days post-infection (equivalent to the 12^th^ to 16^t^^h^ days of egg hatching) (Figure 1A). Subsequently, we isolated and purified primary type II pneumocytes from chicken embryos. As expected, the qPCR results showed that let-7d expression was also significantly inhibited in MG-infected CP-II cells (Figure 1B).

### Downregulated let-7d inhibits pMGA1.2 expression

Adhesion is a necessary step for MG to establish an infection in host cells. The main surface adhesion protein of the MG-HS strain is pMGA1.2, which interacts with apolipoprotein on host cells and promotes the rapid spread of MG-HS in host tissues [6]. To investigate the regulatory effect of let-7d on MG-HS infection, we measured the level of pMGA1.2 by qPCR and Western blotting. Our results demonstrated that both the mRNA and protein levels of pMGA1.2 were significantly increased in the MG-HS-infected CP-II cells. Additionally, overexpression of let-7d further increased the level of pMGA1.2 in CP-II cells, while the let-7d inhibitor significantly inhibited the level of pMGA1.2 (Figure 1C, D).

### Downregulated let-7d inhibits the inflammatory response

Cytokines play a crucial role in innate immunity. To further investigate the regulatory effect of let-7d on innate immunity in MG infection, we measured the levels of inflammatory cytokines, including IL-6, IL-1β, IL-10, and TNF-α, using ELISAs. Our results showed that the expression levels of proinflammatory cytokines (IL-6, IL-1β, and TNF-α) were significantly increased in the MG-infected CP-II cells (*P* < 0.05). Interestingly, the expression levels of TNF-α, IL-1β, and IL-6 were significantly higher in the let-7d overexpression group [mimics (MG+)] than in the negative control group [NC (MG+)], while the results were opposite in the let-7d inhibition group [inhibitor (MG+)] (*P* < 0.05) (Figure 1E). However, we observed an opposite trend in the anti-inflammatory factor IL-10 compared to the proinflammatory factors. These findings suggest that MG infection can induce cellular inflammation, and the low expression of let-7d may inhibit the production of inflammatory cytokines to mitigate inflammation.

### Let-7d contributes to innate immune regulation by targeting MPK1

**Figure 2:**
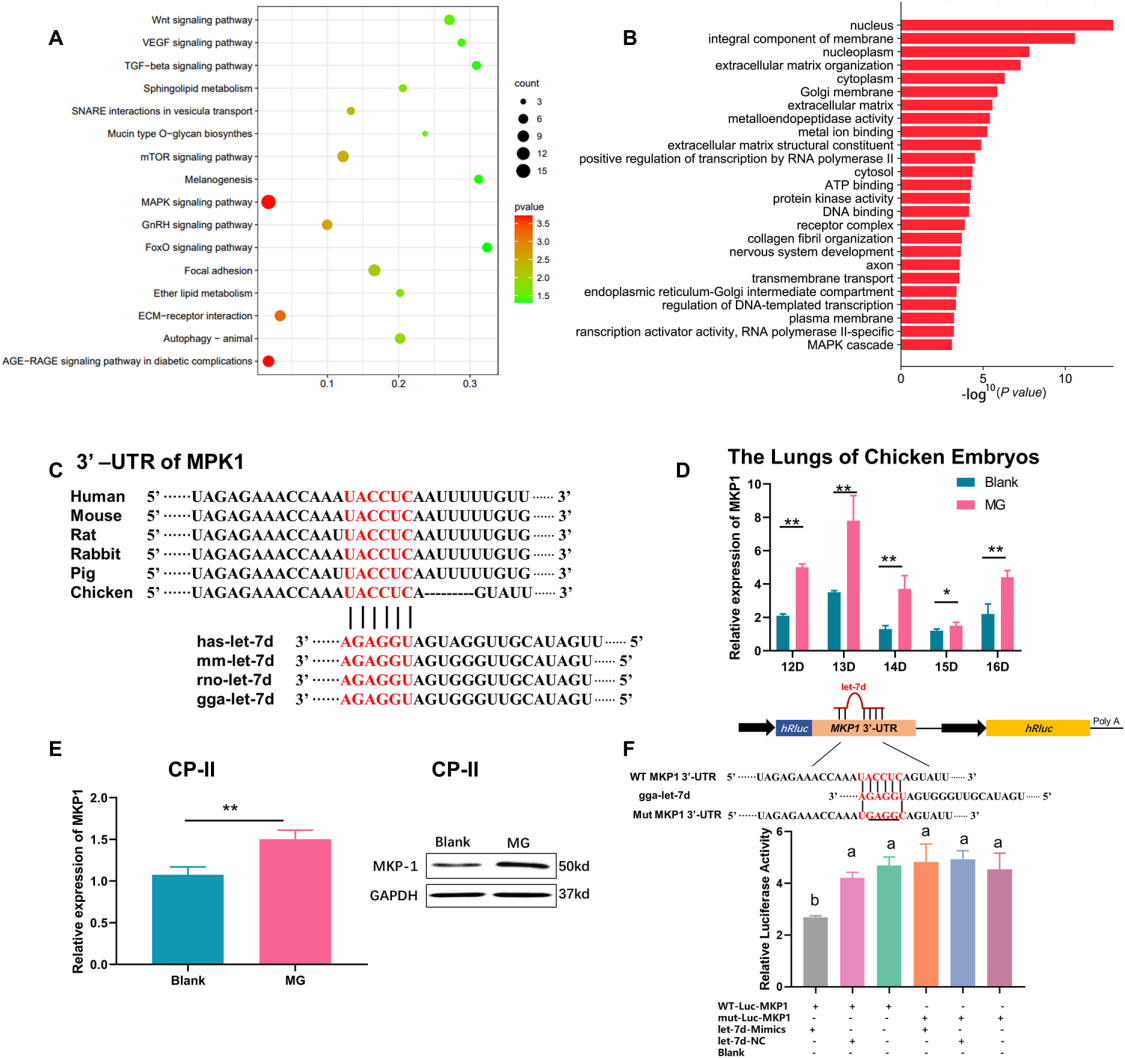
**Let-7d is involved in the regulation of MAPK by targeting MKP1.** **A** KEGG pathway analysis of let-7d target genes. **B** GO term analysis of let-7d target genes. **C** Conservation of the let-7d target sequence in the MKP1 3’-UTR among different species and conservation of the let-7d sequence among different species. **D** The relative level of MKP1 in chicken embryonic lungs. **E** The mRNA and protein levels of MKP1 in CP-II cells. **F **The dual-luciferase reporter assay was performed in CP-II cells. The ratio of Renilla activity: Firefly activity represents luciferase activity. The data are presented as the means ± SDs (*n* = 4). Different lowercase letters represent *p* < 0.05. *p < 0.05, **p < 0.01.

miRNAs regulate gene expression by binding to mRNA transcripts, leading to their degradation or translational inhibition. Therefore, the present study aimed to investigate the targets of let-7d involved in innate immunity to elucidate the molecular mechanisms underlying the inhibition of MG adhesion. Kyoto Encyclopedia of Genes and Genomes (KEGG) pathway and Gene Ontology (GO) analyses of let-7d target genes revealed its involvement in multiple innate immune-related signalling pathways, including the MAPK signalling pathway (Figures 2A, B). Bioinformatics analysis revealed a complementary binding site for let-7d’s seed region in the 3’-UTR of MKP1 mRNA, an endogenous negative regulator of MAPK signalling that is conserved across species (Figure 2C). Moreover, MKP1 expression was significantly upregulated in both MG-infected chicken embryonic lung tissue and CP-II cells, in contrast to the downregulation of let-7d expression (Figures 2D, E). To confirm the relationship between let-7d and MKP1, we constructed dual-luciferase reporter vectors containing either the wild-type or mutant 3’-UTR of MKP1. The results showed that let-7d mimics could significantly reduce the luciferase activity of the reporter containing the wild-type 3’-UTR but had no significant effect on the luciferase activity of the mutant 3’-UTR reporter (Figure 2F). This finding indicated that let-7d could be complementarily bound to the 3’-UTR of MKP1.

**Figure 3:**
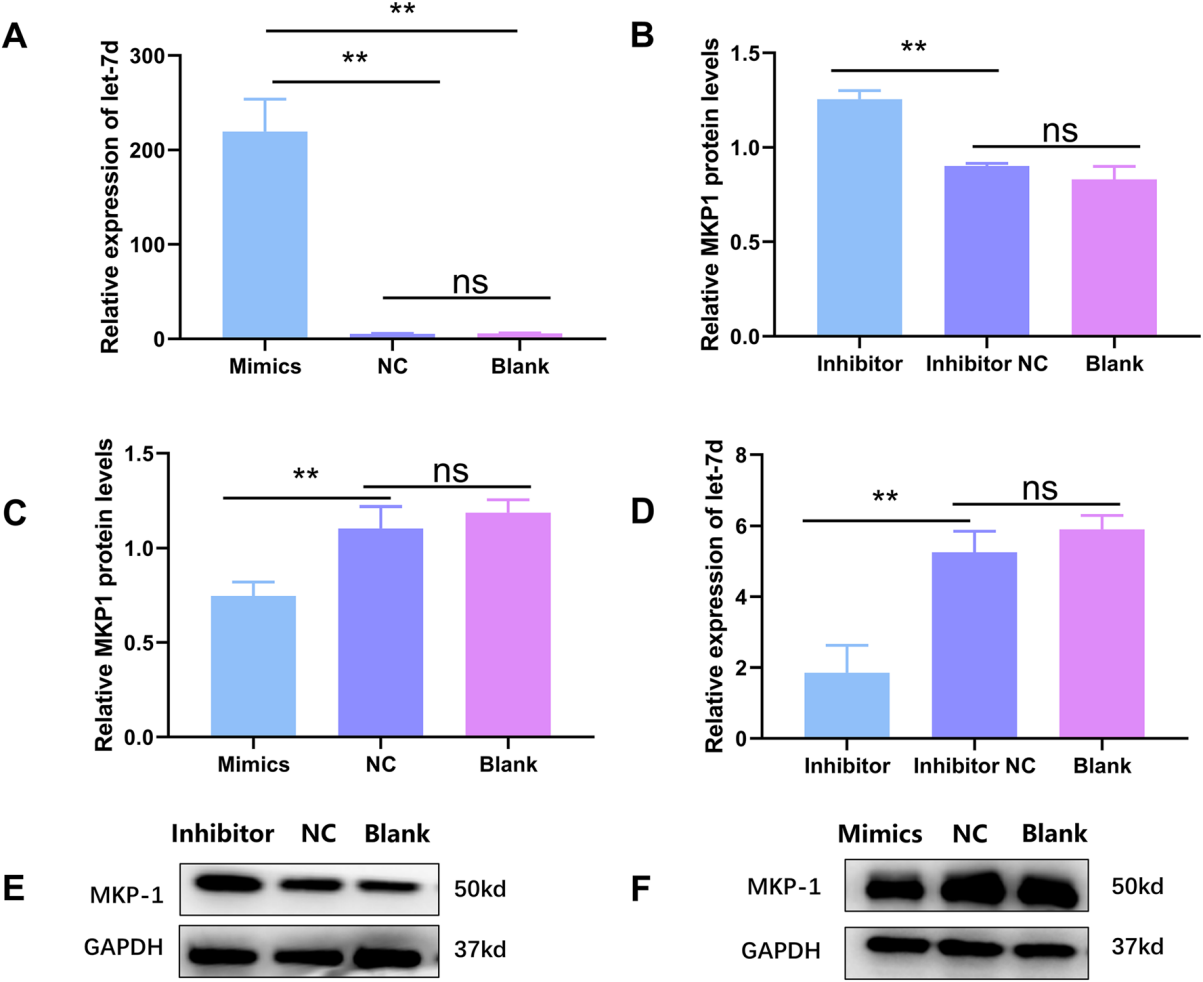
**Let-7d negatively regulates MKP1. A, B**. The levels of let-7d in CP-II cells. **(C-F).** The expression of MKP1 in different treatment groups was detected by qPCR **C, D** and Western blotting **E, F**. The data are the mean ± S.D. of at least 3 independent experiments. *p < 0.05, **p < 0.01. ns > 0.05.

Furthermore, the expression of MKP1 was detected in CP-II cells treated with MG-HS and/or RNA oligonucleotides. As expected, the expression of MKP1 was significantly increased in MG-HS-infected CP-II. Treatment with let-7d mimics significantly inhibited the protein expression of MKP1, while treatment with let-7d inhibitor had the opposite effect (Figure 3). These results confirmed that MKP1 was a direct target of let-7d in CP-II and that its expression was negatively regulated by let-7d. Overall, the study revealed the regulatory effect of let-7d on MG-HS infection and its involvement in the innate immune response through the targeting of MKP1 in the MAPK signalling pathway.

### The MAPK signalling pathway is activated by let-7d

**Figure 4:**
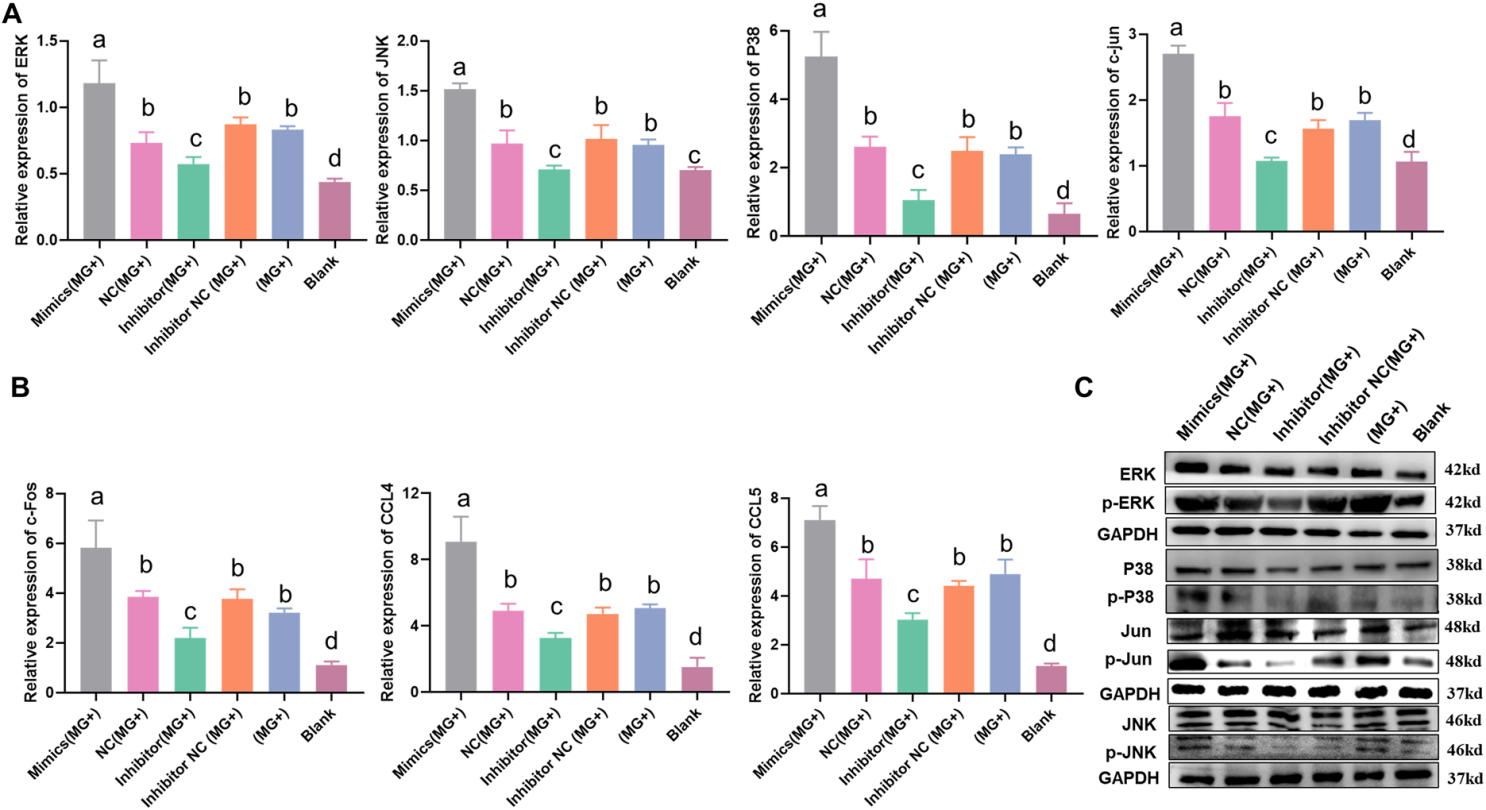
**Let-7d activates the MAPK pathway. A, C** Relative mRNA expression of p38, ERK, jun and JNK. GAPDH was used as the internal quantitative control gene. β-actin and GAPDH served as the loading controls. **B** Effects of gga-let-7d on MAPK pathway downstream related genes in CP-II. RT‒qPCR was used to detect the relative expression of mRNA. GAPDH was used as the internal quantitative control gene. The data are presented as the means ± SDs. One-way ANOVA was used to analyse significant differences (different lowercase letters represent *p* < 0.05).

The above results confirmed that let-7d can directly target the MKP1 gene. To investigate whether let-7d can regulate the MAPK pathway, we detected MAPK signalling pathway-related genes, including P38, ERK, JNK, and c-jun. CP-II cells were transfected with synthesized RNA oligonucleotides and then challenged with 100 µL of MG-HS (1 × 10^9^ CCU/mL) for 12 h. RT‒qPCR was used to measure the relative mRNA expression, while WB was used to detect the protein levels of MAPK pathway-related genes. Additionally, Western blotting was performed to assess the protein levels of p38, P-p38, JNK, P-JNK, ERK, and P-ERK in different treatment groups. Our findings revealed that the overexpression of let-7d in MG-HS-infected CP-II cells significantly increased the mRNA levels of P38, ERK, JNK, and c-jun. Furthermore, WB results showed that the expression of P38, ERK, JNK, and c-jun was noticeably increased by upregulated let-7d, but there was little effect on their total protein in CP-II cells. Conversely, the levels of P38, ERK, JNK, and c-jun were markedly decreased by the let-7d inhibitor but not total protein (Figures 4A and C). Additionally, the overexpression of let-7d significantly upregulated the mRNA expression of c-fos, CCL4, and CCL5, while inhibition of let-7d produced the opposite effect (Figure 4B).

### The target gene MKP1 inhibits the MAPK pathway

**Figure 5:**
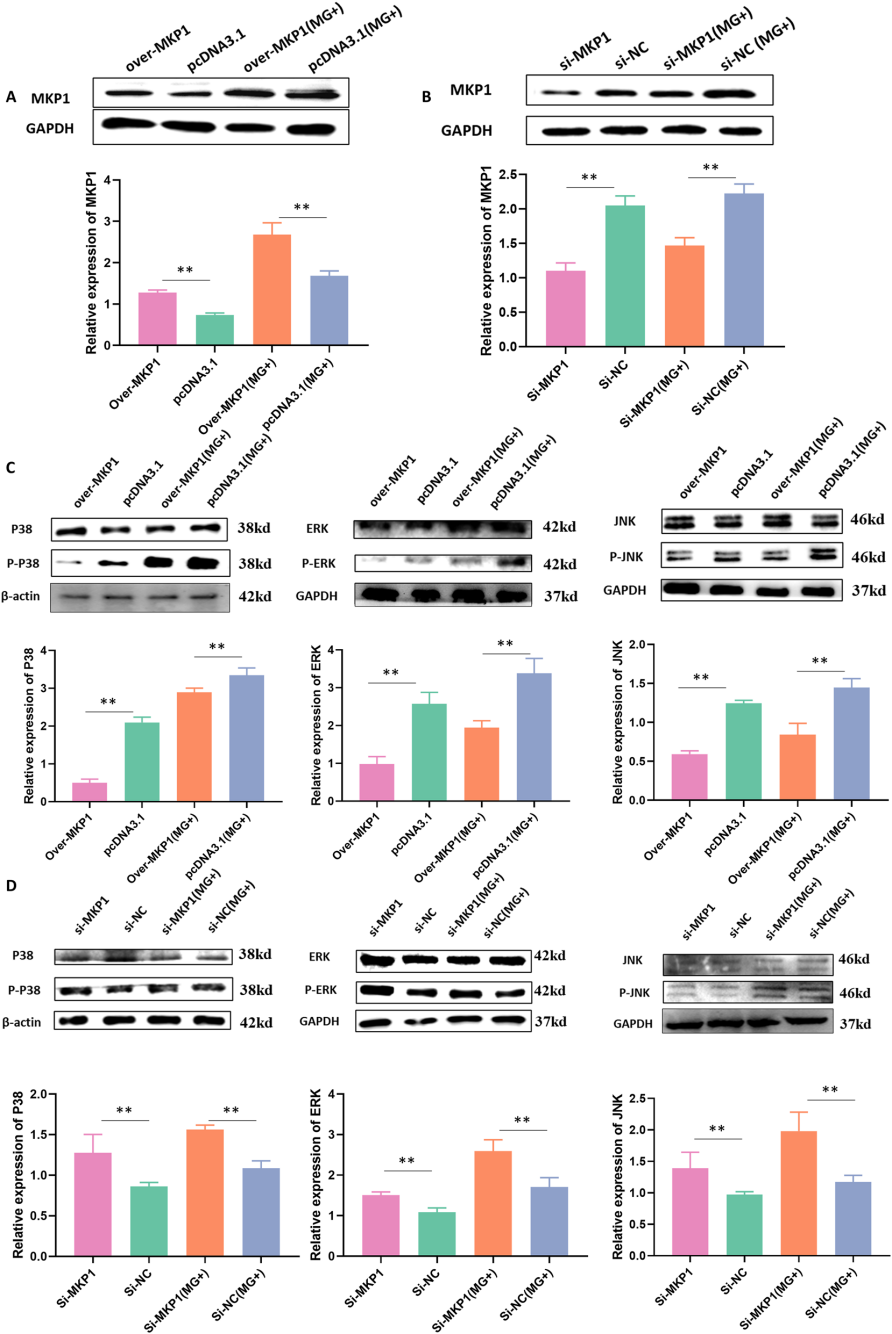
**The regulatory effect of MKP1 on MAPK pathway-related genes.** WB was used to detect protein levels after MKP1 overexpression or knockdown. GAPDH served as the loading control. **A, B** The expression of MKP1. The expression of p38, ERK or JNK in the MKP1 overexpression groups **C** and knockdown groups (**D**). The data are the mean ± S.D. of at least 3 independent experiments. *p < 0.05, **p < 0.01.

To further elucidate the mechanism underlying the regulation of innate immunity by let-7d, we conducted experiments to investigate the effects of MKP1 overexpression or si-MKP1 transfection in CP-II cells infected with MG. As expected, our results showed a significant increase in the level of MKP1 in CP-II cells infected with MG, consistent with our previous findings. We confirmed the knockdown and overexpression of MKP1 in Figures 5A and B. Furthermore, we observed that overexpression of MKP1 resulted in a significant decrease in the levels of p38, ERK, and JNK, whereas their levels were increased by si-MKP1 transfection (Figures 5 C, D).

### MKP1 participates in innate immune regulation

**Figure 6:**
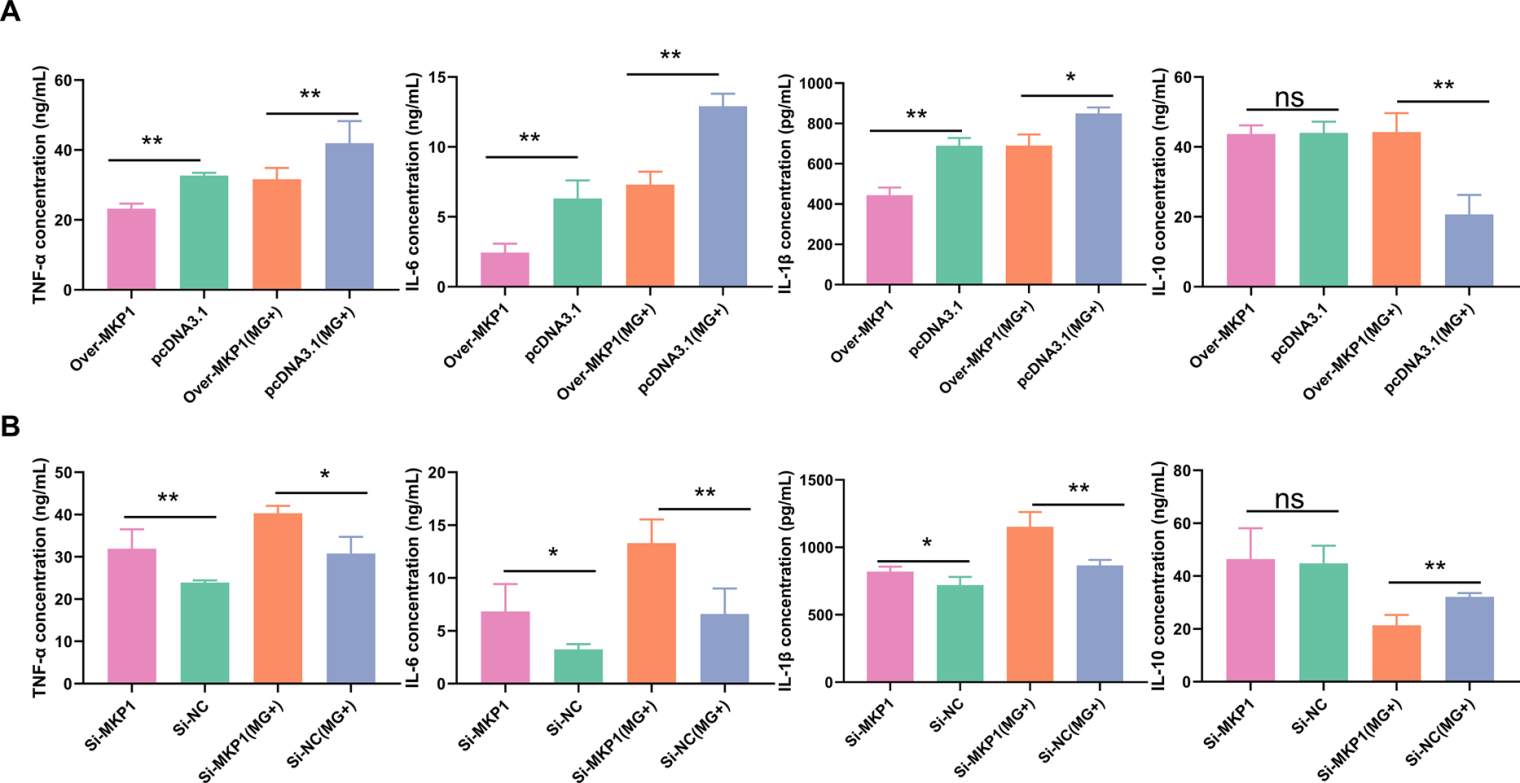
**MKP1 regulates inflammatory cytokine production in CP-II.** CP-II cells were transfected with pcDNA3.1-MKP1 (over MKP1), empty vector (pcDNA3.1), Si-MKP1 or Si-NC for 24 h and then infected with 200 µL of MG-HS. The relative protein expression of inflammatory cytokines in different treatment groups was detected by ELISA kits. The data are presented as the means ± SDs (*n* = 4). * *p* < 0.05, ** *p* < 0.01. ns > 0.05.

An ELISA kit was used to detect the levels of immune factors, including IL-6, IL-1β, IL-10, and TNF-α (Figure 6). The results showed that MG infection significantly induced the release of proinflammatory cytokines (including IL-6, IL-1*β*, and TNF-*α*) but suppressed the release of the anti-inflammatory cytokine IL-10 compared to those of the uninfected group. To investigate the role of MKP1 in regulating inflammatory responses, we examined the effects of MKP1 overexpression and deletion on cytokine production. Our results showed that overexpression of MKP1 significantly inhibited the production of proinflammatory factors (including IL-6, IL-1*β*, and TNF-*α*), while deletion of MKP1 had the opposite effect. Notably, differential expression of MKP1 in the uninfected group did not affect IL-10 expression. However, in the MG-infected group, MKP1 alleviated the inhibitory effect of MG on IL-10 (Figure 6).

### MPK1 exerts proproliferative and antiapoptotic effects on CP-II

**Figure 7:**
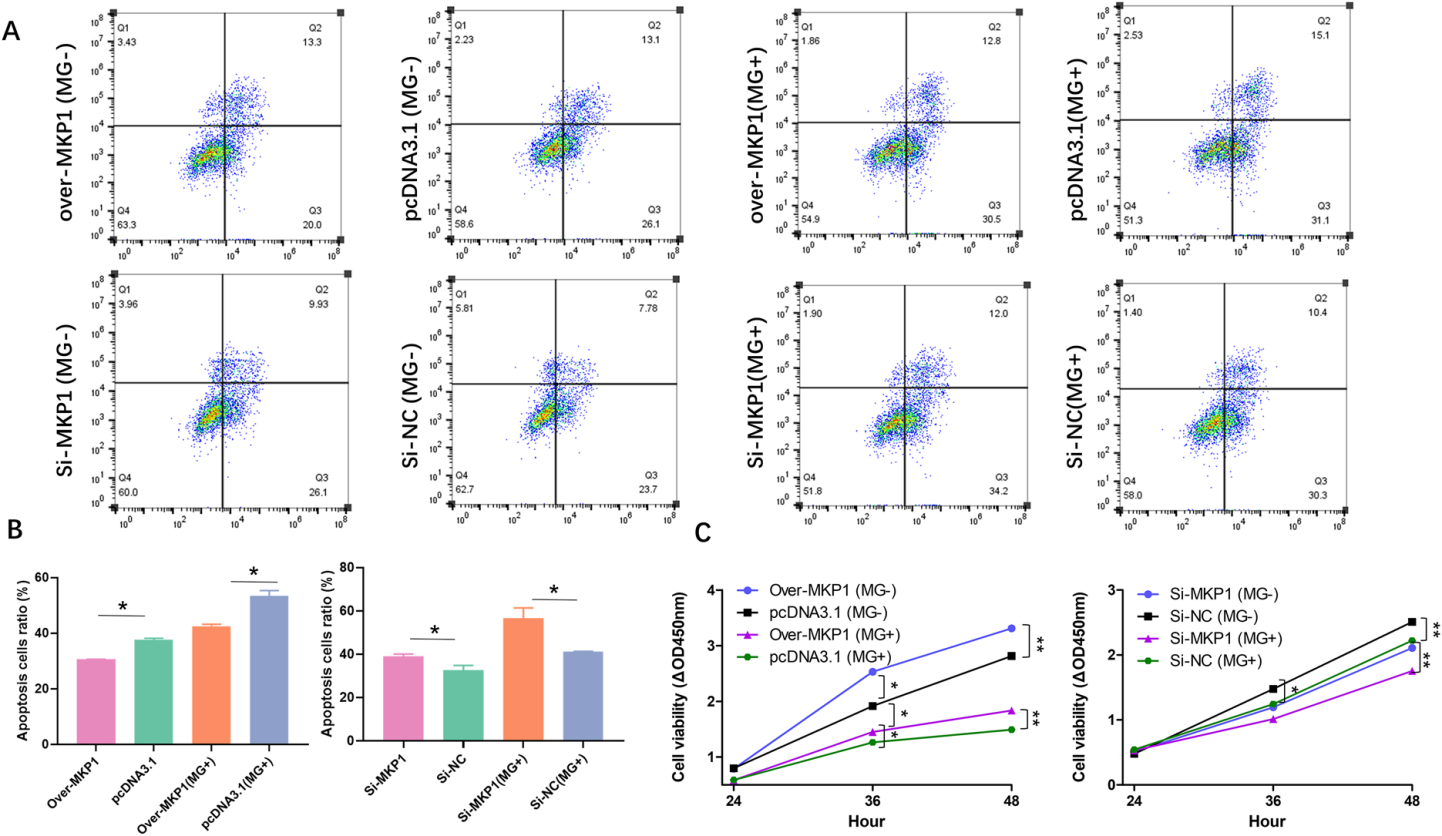
**The effect of MKP1 on CP-II cell proliferation and apoptosis.** CP-II cells were transfected with pcDNA3.1-MKP1 (over MKP1), empty vector (pcDNA3.1), Si-MKP1 or Si-NC for 24 h and then infected with 200 µL of MG-HS. Overexpression of MKP1 was denoted as Over-MKP1; the negative control for MKP1 overexpression was denoted as pcDNA3.1; the MKP1 inhibitor was denoted as Si-MKP1; and the negative control for MKP1 inhibitor was denoted as Si-NC. A CCK-8 kit was used to detect cell proliferation at different times (**A, B**). After 48 h of treatment, the apoptotic cell ratio was analysed by flow cytometry. (**C**). A CCK-8 kit was used to detect cell proliferation at different times. All data from the experiments carried out independently three times are shown as the mean value ± SD, and * *p* < 0.05, ** *p* < 0.01 indicated significant differences.

To further explore how let-7d regulates the anti-MG infection role of MPK1, we manipulated MPK1 expression by knockdown or overexpression. Cell proliferation at different time points was detected using the CCK-8 kit, and CP-II cells were stained with Annexin V-PI and analysed using a flow cytometer. The results indicated that MPK1 overexpression significantly promoted cell proliferation and inhibited apoptosis in CP-II cells, whereas MPK1 knockdown had the opposite effect (Figure 7).

### Downregulated let-7d increases cell proliferation and inhibits apoptosis by targeting MKP1

**Figure 8:**
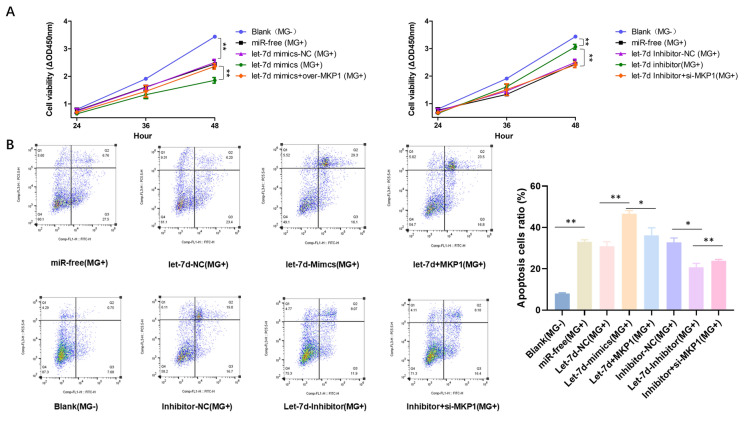
**The effect of let-7d on CP-II cell proliferation and apoptosis.** CP-II cells were transfected with let-7d 50 nM mimics, 100 nM inhibitors, or their respective controls and infected with 100 µL of MG-HS (1 × 10^9^ CCU/mL). Then, a CCK-8 kit was used to detect cell proliferation at different times (**A**). After 48 h of treatment, the CP-II cells were stained with Annexin V– PI and analysed by flow cytometry. The apoptotic cell ratio is shown in **(B)**. All data from the experiments carried out independently three times are shown as the mean value ± SD, and * *p* < 0.05, ** *p* < 0.01 indicated significant differences.

CCK-8 and flow cytometry results showed that after MG infection of CP-II cells, cell proliferation was significantly reduced and the apoptosis rate was significantly increased compared with those of the normal group. Overexpression of let-7d had a significant antiproliferative and proapoptotic effect on CP-II cells, and this effect was partially inhibited by MKP1. Knockout of let-7d showed that the reduction in let-7d promoted cell proliferation and inhibited cell apoptosis, which could be partially mitigated by MPK1 siRNA. These findings provide further evidence that let-7d regulates the proliferation and apoptosis of MG-infected CP-II cells by targeting MKP1 (Figures 8A and B).

## Discussion

After a pathogen infection, the innate immunity of the host is the first line of defence against pathogens. It is the primary method to fight pathogenic infections in chick embryos with weak immune systems [26]. MG is a common causative agent of respiratory infections in the poultry industry that can be spread horizontally and vertically, making it difficult to eliminate the pathogens that typically accompany the eggs laid by infected chickens [27]. For prevention and control of the spread of MG and development of a vaccine against MG, it is essential to understand the innate immunity mechanism of chick embryos against MG. In previous sequencing data, we found a significant downregulation of let-7d in MG-infected chick embryos. We isolated and purified chicken primary type II pneumocytes (CP-II) to mimic the chicken embryo innate immunity process and found that low expression of let-7d could inhibit the expression of MG-HS adhesion protein (pMGA1.2) (Figures 1A–D), which is essential for the MG-induced inflammatory response [6, 21]. Our data showed that let-7d may be involved in the innate immune mechanism of resistance to MG infection. However, numerous prediction software programs failed to show that let-7d contained a direct binding site to the pMGA1.2 protein sequence. It is predicted that let-7d does not directly inhibit MG adhesion but may be engaged in the regulation of pMGA1.2 through the modulation of innate immune-related factors.

Although the inflammatory response is the first response of the innate immune system against pathogens, an excessive immune response to pathogen invasion can cause a “cytokine storm” that severely damages host organs and even leads to death. Numerous studies have shown that miRNAs are involved in the regulation of host cytokine storms upon pathogen invasion. MiR-223 attenuates acute inflammation in the lungs of mice caused by *Streptococcus agalactiae* [28]; inhibition of miRNA-34a promotes M2 macrophage polarization and ameliorates LPS-induced lung injury by targeting Kruppel-like factor 4 (KLF4) [29]; and miR-126-5p regulates host innate immune responses by suppressing the host inflammatory factor storm [30]. In the present study, we found that let-7d was able to suppress the MG-triggered cytokine storm to regulate the host immune response (Figure 1E). This negative regulation effectively avoids damage to the host from an excessively strong immune response.

Our findings suggest that let-7d may play a role in innate immunity by regulating the MAPK pathway through targeting the MKP1 gene (Figure 2). MAPKs are a family of serine/threonine protein kinases that direct cellular responses to various stimuli, including pathogens and inflammatory cytokines such as TNF-α and IL-6. These stimuli regulate immunoreaction, cell proliferation, cell survival, and apoptosis [31, 32]. Numerous studies have shown that MAPKs can be activated by pathogens, and activated MAPKs phosphorylate various proteins, including transcription factors, leading to the regulation of inflammatory responses [33]. The inflammatory response is a major pathological feature of MG infection, causing chronic respiratory disease in chickens [34]. In addition, our previous results showed that the MAPK pathway might be a critical regulatory route for MG infection [11]. Our results showed that low expression of let-7d activated the MAPK pathway, thereby inhibiting the MG-induced inflammatory response to anti-MG infection (Figures 4, 6). This finding is consistent with other researchers who have observed that let-7d can play an important role in resistance to pathogen infection and immune regulation [35]. Perhaps precisely for the specificity of let-7, as described in this paper, let-7d plays a critical regulatory role in CP-II cells infected with MG.

MKP1, a major factor in the negative regulation of p38, JNK and ERK, inhibits phosphorylation of the MAPK family [15]. Phosphorylation or dephosphorylation of p38, JNK, and ERK regulates the activation or inactivation of downstream inflammatory and apoptotic pathways [36]. MKP1 has been reported to be involved in immunoregulation, the inflammatory response, and apoptosis through the regulation of the MAPK signalling pathway in the pathogenesis of several diseases [37]. Downregulation of MKP1 can promote the proliferation of tumours at advanced stages of tumorigenesis by enhancing the ERK/MAPK signalling pathway [15]. Knockdown of the MKP1 gene promotes the production of proinflammatory cytokines (TNF-*α* and IL-6) and chemokines (MIP-1α/CCL3) in mouse models of *Escherichia coli* and *S. aureus* infection [38]. In many cancer studies, MKP1 inhibits tumour progression by inhibiting ERK phosphorylation. JNK phosphorylation has been reported to upregulate the expression of IL-6 and TNF-*α* [39]. More importantly, the mechanism of action of many commonly used anti-disease drugs is through the modulation of MKP1 [40]. NSC 95,397 exerts antiproliferative and proapoptotic effects on colon cancer cells through inhibition of MKP-1 activity and subsequent activation of ERK1/2 [41]. Sinomenine (SIN) was found to attenuate LPS-induced inflammatory damage in HaCaT cells by regulating MKP-1 [42]. In addition, numerous studies have shown that altered innate immune responses are associated with disorders of cell proliferation and apoptotic signalling. Consistent with these results, our results showed that MKP1, a target gene of let-7d, could be significantly upregulated after infection with MG (Figure 2). Overexpression of MKP1 inhibited the MAPK signalling pathway, promoted proliferation and inhibited apoptosis by significantly downregulating the expression of the MAPK pathway genes P38, JNK and ERK (Figures 5,  7). Moreover, it could resist MG infection by decreasing the release of TNF-*α*, IL-1*β* and IL-6 but elevating the secretion of IL-10 (Figure 6).

In conclusion, our results demonstrate that let-7d is involved in the innate immune response of chick embryos against MG infection by regulating the MAPK pathway through targeting MKP1. The findings shed light on the regulatory mechanisms of innate immunity against MG and provide a potential target for the development of new anti-MG therapies. Further investigation is needed to fully understand the complex interactions among let-7d, the MAPK pathway, and innate immunity in MG infection. Our study provides a foundation for future studies on the roles of miRNAs in innate immunity and host‒pathogen interactions.

## Data Availability

The datasets analysed in the present study are available from the corresponding author on reasonable request.

## References

[1] Wang Y, Wang L, Luo R, Sun Y, Zou M, Wang T, Guo Q, Peng X (2022). Glycyrrhizic acid against *Mycoplasma gallisepticum*-induced inflammation and apoptosis through suppressing the MAPK pathway in chickens. J Agric Food Chem.

[2] Awad NFS, Abd El-Hamid MI, Hashem YM, Erfan AM, Abdelrahman BA, Mahmoud HI (2019). Impact of single and mixed infections with *Escherichia coli* and *Mycoplasma gallisepticum* on Newcastle disease virus vaccine performance in broiler chickens: an in vivo perspective. J Appl Microbiol.

[3] Fürnkranz U, Siebert-Gulle K, Rosengarten R, Szostak MP (2013). Factors influencing the cell adhesion and invasion capacity of *Mycoplasma gallisepticum*. Acta Vet Scand.

[4] Sun Y, Wang Y, Zhao Y, Zou M, Peng X (2021). Exosomal miR-181a-5p reduce *Mycoplasma gallisepticum* (HS strain) infection in chicken by targeting PPM1B and activating the TLR2-mediated MyD88/NF-κB signaling pathway. Mol Immunol.

[5] Wang Y, Wang L, Hu F, Zou M, Luo R, Sun Y, Wang T, Guo Q, Peng X (2022). Extracellular HMGB1 as inflammatory mediator in the progression of *Mycoplasma Gallisepticum* infection. Cells.

[6] Hu F, Zhao C, Bi D, Tian W, Chen J, Sun J, Peng X (2016). *Mycoplasma gallisepticum* (HS strain) surface lipoprotein pMGA interacts with host apolipoprotein A-I during infection in chicken. Appl Microbiol Biotechnol.

[7] Wang Y, Han Y, Wang L, Zou M, Sun Y, Sun H, Guo Q, Peng X (2022). *Mycoplasma gallisepticum* escapes the host immune response via gga-miR-365-3p/SOCS5/STATs axis. Vet Res.

[8] Mahdizadeh S, Sansom FM, Lee SW, Browning GF, Marenda MS (2020). Targeted mutagenesis of *Mycoplasma gallisepticum* using its endogenous CRISPR/Cas system. Vet Microbiol.

[9] Platt A, Wetzler L (2013). Innate immunity and vaccines. Curr Top Med Chem.

[10] Kim JK, Kim TS, Basu J, Jo EK (2017). MicroRNA in innate immunity and autophagy during mycobacterial infection. Cell Microbiol.

[11] Zhao Y, Hou Y, Zhang K, Yuan B, Peng X (2017). Identification of differentially expressed miRNAs through high-throughput sequencing in the chicken lung in response to *Mycoplasma gallisepticum* HS. Comp Biochem Physiol Part D Genomics Proteomics.

[12] Zhi L, Yu Y, Li X, Wang D, Wang D (2017). Molecular control of innate immune response to *Pseudomonas aeruginosa* infection by intestinal let-7 in *Caenorhabditis elegans*. PLoS Pathog.

[13] Teng GG, Wang WH, Dai Y, Wang SJ, Chu YX, Li J (2013). Let-7b is involved in the inflammation and immune responses associated with *Helicobacter pylori* infection by targeting toll-like receptor 4. PLoS One.

[14] Chen XM, Splinter PL, O’Hara SP, LaRusso NF (2007). A cellular micro-RNA, let-7i, regulates toll-like receptor 4 expression and contributes to cholangiocyte immune responses against *Cryptosporidium parvum* infection. J Biol Chem.

[15] Kirk SG, Samavati L, Liu Y (2020). MAP kinase phosphatase-1, a gatekeeper of the acute innate immune response. Life Sci.

[16] Candas D, Li JJ (2015). MKP1 mediates resistance to therapy in HER2-positive breast tumors. Mol Cell Oncol.

[17] Turpeinen T, Nieminen R, Taimi V, Heittola T, Sareila O, Clark AR, Moilanen E, Korhonen R (2011). Dual specificity phosphatase 1 regulates human inducible nitric oxide synthase expression by p38 MAP kinase. Mediators Inflamm.

[18] Diaz-Cañestro C, Reiner MF, Bonetti NR, Liberale L, Merlini M, Wüst P, Amstalden H, Briand-Schumacher S, Semerano A, Giacalone G, Sessa M, Beer JH, Akhmedov A, Lüscher TF, Camici GG (2019). AP-1 (activated protein-1) transcription factor JunD regulates ischemia/reperfusion brain damage via IL-1β (interleukin-1β). Stroke.

[19] Zhao Y, Fu Y, Zou M, Sun Y, Yin X, Niu L, Gong Y, Peng X (2020). Analysis of deep sequencing exosome-microRNA expression profile derived from CP-II reveals potential role of gga-miRNA-451 in inflammation. J Cell Mol Med.

[20] Bi D, Ji X (1988). The isolation and identification of the *Mycoplasma gallisepticum*. Acta Vet Zootechnol Sin.

[21] Bi D, Q X (1997). Study on pathogenicity of HS strain *Mycoplasma gallisepticum*. Chin J Anim Poult Infect Dis.

[22] Zou M, Yang W, Niu L, Sun Y, Luo R, Wang Y, Peng X (2020). Polydatin attenuates *Mycoplasma gallisepticum* (HS strain)-induced inflammation injury via inhibiting the TLR6/ MyD88/NF-κB pathway. Microb Pathog.

[23] Wang Y, Liang Y, Hu F, Sun Y, Zou M, Luo R, Peng X (2022). Chinese herbal formulae defend against *Mycoplasma gallisepticum* infection. J Integr Agric.

[24] Livak KJ, Schmittgen TD (2001). Analysis of relative gene expression data using real-time quantitative PCR and the 2(-Delta Delta C(T)) method. Methods.

[25] Yin X, Wang Y, Sun Y, Han Y, Sun H, Zou M, Luo R, Peng X (2021). Down-regulated gga-miR-223 inhibits proliferation and induces apoptosis of MG-infected DF-1 cells by targeting FOXO3. Microb Pathog.

[26] Alkie TN, Yitbarek A, Hodgins DC, Kulkarni RR, Taha-Abdelaziz K, Sharif S (2019). Development of innate immunity in chicken embryos and newly hatched chicks: a disease control perspective. Avian Pathol.

[27] Wang Y, Tong D, Sun Y, Sun H, Liu F, Zou M, Luo R, Peng X (2021). DF-1 cells prevent MG-HS infection through gga-miR-24-3p/RAP1B mediated decreased proliferation and increased apoptosis. Res Vet Sci.

[28] Deny M, Romano M, Denis O, Casimir G, Chamekh M (2020). Progressive control of Streptococcus agalactiae-induced innate inflammatory response is associated with time course expression of microRNA-223 by neutrophils. Infect Immun.

[29] Khan MJ, Singh P, Dohare R, Jha R, Rahmani AH, Almatroodi SA, Ali S, Syed MA (2020). Inhibition of miRNA-34a promotes M2 macrophage polarization and improves LPS-induced lung injury by targeting Klf4. Genes.

[30] Wang J, Cheng Y, Wang L, Sun A, Lin Z, Zhu W, Wang Z, Ma J, Wang H, Yan Y, Sun J (2022). Chicken mir-126-5p negatively regulates antiviral innate immunity by targeting TRAF3. Vet Res.

[31] Mai L, Zhu X, Huang F, He H, Fan W (2020). p38 mitogen-activated protein kinase and pain. Life Sci.

[32] Korhonen R, Moilanen E (2014). Mitogen-activated protein kinase phosphatase 1 as an inflammatory factor and drug target. Basic Clin Pharmacol Toxicol.

[33] Cui L, Zheng Y, Wang H, Dong J, Li J, Song Q, Qian C, Li J (2020). Cortisol inhibits the *Escherichia coli*-induced endometrial inflammatory response through NF-κB and MAPK pathways in postpartum goats. Anim Reprod Sci.

[34] Ishfaq M, Zhang W, Ali Shah SW, Wu Z, Wang J, Ding L, Li J (2020). The effect of *Mycoplasma gallisepticum* infection on energy metabolism in chicken lungs: through oxidative stress and inflammation. Microb Pathog.

[35] Gilles ME, Slack FJ (2018). Let-7 microRNA as a potential therapeutic target with implications for immunotherapy. Expert Opin Ther Targets.

[36] Sha J, Feng X, Chen Y, Zhang H, Li B, Hu X, Fan H (2019). Dexmedetomidine improves acute stress-induced liver injury in rats by regulating MKP-1, inhibiting NF-κB pathway and cell apoptosis. J Cell Physiol.

[37] Liu D, Du J, Sun J, Li M (2021). Parathyroid hormone-related protein inhibits nitrogen-containing bisphosphonate-induced apoptosis of human periodontal ligament fibroblasts by activating MKP1 phosphatase. Bioengineered.

[38] Frazier WJ, Wang X, Wancket LM, Li XA, Meng X, Nelin LD, Cato AC, Liu Y (2009). Increased inflammation, impaired bacterial clearance, and metabolic disruption after gram-negative sepsis in Mkp-1-deficient mice. J Immunol.

[39] Yan C, Deng C, Liu X, Chen Y, Ye J, Cai R, Shen Y, Tang H (2018). TNF-α induction of IL-6 in alveolar type II epithelial cells: contributions of JNK/c-Jun/AP-1 element, C/EBPδ/C/EBP binding site and IKK/NF-κB p65/κB site. Mol Immunol.

[40] Yang J, Sun L, Han J, Zheng W, Peng W (2019). DUSP1/MKP-1 regulates proliferation and apoptosis in keratinocytes through the ERK/Elk-1/Egr-1 signaling pathway. Life Sci.

[41] Dubey NK, Peng BY, Lin CM, Wang PD, Wang JR, Chan CH, Wei HJ, Deng WP (2018). NSC 95397 suppresses proliferation and induces apoptosis in colon cancer cells through MKP-1 and the ERK1/2 pathway. Int J Mol Sci.

[42] Liu S, Man Y, Zhao L (2018). Sinomenine inhibits lipopolysaccharide-induced inflammatory injury by regulation of miR-101/MKP-1/JNK pathway in keratinocyte cells. Biomed Pharmacother.

